# Ambient Particulate Matter Induces Interleukin-8 Expression through an Alternative NF-κB (Nuclear Factor-Kappa B) Mechanism in Human Airway Epithelial Cells

**DOI:** 10.1289/ehp.1103594

**Published:** 2011-06-10

**Authors:** Robert Silbajoris, Alvaro R. Osornio-Vargas, Steven O. Simmons, William Reed, Philip A. Bromberg, Lisa A. Dailey, James M. Samet

**Affiliations:** 1 Environmental Public Health Division, National Health and Environmental Effects Research Laboratory, U.S. Environmental Protection Agency, Research Triangle Park, North Carolina, USA; 2 Instituto Nacional de Cancerologia, Mexico City, Mexico; 3 Integrated Systems Toxicology Divisions, National Health and Environmental Effects Research Laboratory, U.S. Environmental Protection Agency, Research Triangle Park, North Carolina, USA; 4 Center for Environmental Medicine, Asthma and Lung Biology, University of North Carolina at Chapel Hill, Chapel Hill, North Carolina, USA

**Keywords:** human airway epithelium, interleukin-8, NF-κB, particulate matter, signal transduction

## Abstract

Background: Exposure to ambient air particulate matter (PM) has been shown to increase rates of cardiopulmonary morbidity and mortality, but the underlying mechanisms are still not well understood.

Objective: We examined signaling events involved in the expression of the inflammatory gene interleukin-8 (*IL-8*) in human airway epithelial cells (HAECs) exposed to ambient PM collected in an urban area of Mexicali, Mexico.

Methods: We studied *IL-8* expression and regulatory signaling pathways in cultured HAECs exposed to Mexicali PM suspended in media for 0–4 hr.

Results: Exposure resulted in a dose-dependent, 2- to 8-fold increase in *IL-8* mRNA expression relative to controls. PM exposure induced *IL-8* transcriptional activity in BEAS-2B cells that was dependent on the nuclear factor-kappa B (NF-κB) response element in the *IL-8* promoter. Chromatin immunoprecipitation (ChIP) assays showed a 3-fold increase in binding of the p65 (RelA) NF-κB isoform to the *IL-8* promoter sequence in HAECs exposed to PM. Western blot analyses showed elevated levels of phosphorylation of p65 but no changes in IκBα phosphorylation or degradation. *IL-8* expression was blunted in a dose-dependent manner in BEAS-2B cells transduced with a lentivirus encoding a dominant negative p65 mutant in which phosphorylation sites were inactivated.

Conclusion: Taken together, these findings show that the increase in *IL-8* mRNA expression in HAECs exposed to PM_10_ (PM ≤ 10 μm in aerodynamic diameter) is mediated through an NF-κB–dependent signaling mechanism that occurs through a pathway involving direct phosphorylation of the transcription factor p65 in the absence of IκBα degradation. These data show that exposure to PM_10_ in ambient air can induce inflammatory responses by activating specific signaling mechanisms in HAECs.

Epidemiological studies have demonstrated a positive association between levels of ambient particulate matter (PM) and mortality and morbidity (Dockery et al. 1993; Samet et al. 2000). Health effects associated with PM exposure include decreased pulmonary function, exacerbation of respiratory disease, and increased acute cardiovascular mortality (Pope 2000). In addition to PM concentration on a mass and number basis, physicochemical properties of the particles (e.g., chemical composition, surface area, size) have been proposed as determinants of the adverse effects associated with PM inhalation (Schwarze et al. 2006). However, despite being the subject of more than a decade of study, the toxicological mechanisms underlying the health effects of PM inhalation are not well understood.

Human airway epithelial cells (HAECs) are a primary target for inhaled PM as well as a major source of inflammatory mediators in the lung, including the potent neutrophil chemoattractant *interleukin-8* (*IL-8*) (Strieter 2002), which plays an important role in pathogenic responses in the human lung. Elevated levels of *IL-8* in the lung are a feature of a number of respiratory diseases, including cystic fibrosis, asthma, and chronic obstructive pulmonary disease (Bonfield et al. 1995; Gibson et al. 2001; Yamamoto et al. 1997). Increased expression of *IL-8*, therefore, is a pivotal event in the pulmonary inflammatory response associated with disease states (Harada et al. 1994; Zwahlen et al. 1993) and inhalation of ambient air contaminants (Ghio and Devlin 2001; Krishna et al. 1998; Stenfors et al. 2004).

*IL-8* expression is regulated by transcriptional and posttranscriptional mechanisms under the control of multiple signaling pathways. In addition to a CCAAT box, regulatory elements have been identified in the 5´-flanking region of the *IL-8* gene for multiple transcription factors, including glucocorticoid receptor, hepatocyte nuclear factor-1, interferon regulatory factor-1, activator protein-1 (AP-1), CCAAT/enhancer binding protein β  (C/EBPβ), and nuclear factor-kappa B (NF-κB) (Jung et al. 2002; Mukaida et al. 1994).  In addition, mRNA stabilization can play a role in increased *IL-8* expression (Silbajoris et al. 2009; Sparkman and Boggaram 2004).

The transcription factor NF-κB is the best-characterized regulator of *IL-8* transcription in HAECs. NF-κB functions as a heterogeneous collection of dimers composed of various combinations of five members of the NF-κB family. Classical NF-κB activation involves phosphorylation of the inhibitory subunit IκBα on two N-terminal serine residues by IκB kinases, rapidly followed by ubiquitination and proteosomal degradation of IκBα. The p50–p65 NF-κB heterodimer is thereby released from the complex and translocates to the nucleus, where it binds to the NF-κB response element, such as that found in the promoter region of the *IL-8* gene (Karin and Ben-Neriah 2000). NF-κB–dependent gene transcription is also regulated by serine or threonine phosphorylations or other modifications of the Rel subunits that affect the transcription-activating function of NF-κB (Neumann and Naumann 2007). Thus, NF-κB–dependent gene transcription can be regulated by mechanisms that involve only NF-κB transactivation in the absence of IκB degradation and NF-κB nuclear translocation (Bohuslav et al. 2004; Neumann and Naumann 2007; Zhang et al. 2005). These IκBα-independent pathways can involve posttranslational modifications of NF-κB subunits, including site-specific phosphorylation of p65 (Neumann and Naumann 2007).

To characterize relevant mechanisms that lead to inflammatory responses to PM inhalation, we examined the activation of signaling events involved in *IL-8* expression in a model of human airway epithelium exposed to PM obtained from Mexicali, a city in Mexico that experiences some of the highest PM levels in North America (Osornio-Vargas et al. 2010). Exposure of cultured HAECs to PM ≤ 10 μm in aerodynamic diameter (PM_10_) collected from Mexicali ambient air induced NF-κB–dependent transcriptional expression of IL-8 through an alternate signaling pathway involving direct phosphorylation of p65.

## Materials and Methods

*Reagents and supplies.* We obtained tissue culture media and supplements from Lonza (Walkersville, MD, USA), Costar tissue culture plates from Corning (Corning, NY, USA), human recombinant tumor necrosis factor-α (TNFα) from Peprotech (Rocky Hill, NJ, USA), and MG-132 from Calbiochem (La Jolla, CA, USA). Western blotting supplies were obtained from Bio-Rad Laboratories (Hercules, CA, USA). Common laboratory reagents and supplies were obtained from Sigma (St. Louis, MO, USA) and Fisher Scientific (Raleigh, NC, USA).

*Cell culture.* After receiving informed consent from volunteers, we obtained HAECs from normal adult human volunteers by brush biopsy of the mainstem bronchus during fiberoptic bronchoscopy; this procedure was conducted under a protocol approved by the Committee on the Protection of the Rights of Human Subjects at the University of North Carolina at Chapel Hill. HAECs were cultured as previously described (Silbajoris et al. 2009). Transformed human airway epithelial cells (BEAS-2B subclone S6, passages 60–80) were grown on tissue culture plates in supplemented keratinocyte growth medium. HAECs and BEAS-2B cells were growth factor–starved in unsupplemented media for 15 hr before PM exposure.

*PM.* PM_2.5_ (PM ≤ 2.5 µm in aerodynamic diameter) and PM_10_ size fractions were collected between October 2005 and March 2006 at urban and semiurban locations in Mexicali, Mexico (Osornio-Vargas et al. 2010). For additional methodological details and PM_10_ elemental composition and size, see Supplemental Material, p. 2 and Table 1 (http://dx.doi.org/10.1289/ehp.1103594). PM suspensions were prepared by sonicating the recovered particles in supplement-free basal media in an ultrasonic water bath immediately before cell exposure.

*Plasmid construction and lentiviral vector production.* Lentiviral transfer plasmids for human *IL-8* wild-type (*IL-8*wt) and NF-κB–site mutant (*IL-8*m^–^ NF-κB) luciferase reporters, as well as the NF-κB tandem repeat (NF-κBtr) luciferase reporter, have been previously described (Tal et al. 2010). For additional methodological details, see Supplemental Material, pp. 2–3 (http://dx.doi.org/10.1289/ehp.1103594).

*Reverse-transcription polymerase chain reaction (RT-PCR).* We quantified relative gene expression in HAECs and BEAS-2B cells using RT-PCR as previously described (Tal et al. 2010). For additional methodological details, see Supplemental Material, pp. 3–4 and Table 2 (http://dx.doi.org/10.1289/ehp.1103594).

*IL-8 promoter–reporter activity assay.* BEAS-2B cells grown to 50% confluency were transduced with lentiviral IL-8wt, IL-8m^–^ NF-κB, or NF-κBtr for 72 hr with a multiplicity of infection (MOI; number of viral particles per cell) of 5. Cells were cotransduced with EF1a_EGFP (elongation factor 1-alpha–enhanced green fluorescent protein) to control for transduction efficiency. Transduced cells were changed to supplement-free media overnight and then exposed to Mexicali urban PM_10_ at 40 μg/cm^2^ for 4 hr or to TNFα (20 ng/mL) for 15 min. Because preliminary experiments showed that the presence of particles interfered with optical measurements of luminescence and fluorescence, promoter activity was assessed by measuring firefly luciferase (*fLuc*) mRNA levels in cDNA generated from cell lysates.

*ChIP (chromatin immunoprecipitation) assay.* HAECs were changed to supplement-free media overnight and then exposed to Mexicali urban PM_10_ at 40 μg/cm^2^ for 4 hr or to TNFα (20 ng/mL) for 15 min. We then performed a ChIP assay using the Chromatin Immunoprecipitation Assay kit (Upstate, Charlottesville, VA, USA) according to the manufacturer’s instructions. Briefly, protein–DNA complexes were cross-linked with formaldehyde, cells were lysed and pelleted, and chromatin was sheared by sonication. Equal amounts of DNA were precleared with salmon sperm DNA/protein A agarose, and NF-κB–bound DNA fragments were immunoprecipitated with an antibody against NF-κBp65 (Santa Cruz Biotechnology, Santa Cruz, CA, USA). A sham immunoprecipitation using no antibody was used as a control. The antibody–DNA complex was captured with salmon sperm DNA/protein A agarose, pelleted, and washed. DNA was eluted with TE (Tris–EDTA) buffer, and cross-linking was reversed with 5 M NaCl at 65°C. RT-PCR, employing primers and a fluorescent probe directed against the NF-κB sequence in the *IL-8* gene promoter region, was used to measure percentage of input, by comparing signals of ChIP samples and total input (DNA signal obtained by PCR amplification of samples taken before immunoprecipitation).

*Western blotting.* HAECs were changed to supplement-free media overnight and then exposed to PM_10_ (40 μg/cm^2^), TNFα (20 ng/mL), or media alone. To measure expression levels of phosphorylated IκBα (p-IκBα) we pretreated HAECs with the proteosomal inhibitor MG-132 (20 μM). Protein extracts were subjected to SDS-PAGE. Each resulting blot was blocked with 5% casein or bovine serum albumin and incubated with one of the following antibodies: anti-phosphorylated-NF-κBp65 (anti-p-NF-κBp65; Ser 536), anti-NF-κBp65 (Ser 536), anti-p-IκBα (Cell Signaling, Beverly, MA, USA), anti-IκBα, or β-actin (Santa Cruz Biotechnology). After incubation with horseradish peroxidase (HRP) conjugated goat anti-rabbit IgG and HRP conjugated goat anti-mouse IgG secondary antibodies (Santa Cruz Biotechnology), bands were detected using ECL chemiluminescence reagents (Amersham, Piscataway, NJ, USA) and visualized using a Fujifilm LAS-3000 imaging system (Fujifilm, Stamford, CT, USA).

*Inhibition of* IL-8 *transcription using pTRED-CMV_p65 triple mutant.* BEAS-2B cells grown to 50% confluency were transduced with the lentiviral pTRED-CMV_p65 triple mutant for 72 hr with an MOI of 5 or 10. Cells were changed to supplement-free media overnight and then exposed to Mexicali urban PM_10_ at 40 μg/cm^2^ or media alone for 4 hr. Levels of *IL-8* mRNA in cell lysates were measured using RT-PCR, normalized to levels of glyceraldehyde 3-phosphate dehydrogenase (*GAPDH*) mRNA, and are expressed as fold increases over media control.

*Statistics.* Data are expressed as mean ± SE from at least three independent experiments. We performed data comparisons using one-way analysis of variance followed by Dunnett’s post hoc test for multigroup comparisons. Two-tailed paired or unpaired Student’s *t*-tests were used to evaluate differences between control and treated groups. Where results are presented as relative fold increases, statistics were performed using absolute data. Differences were assessed as significant when *p* ≤ 0.05.

## Results

**Figure 1:**
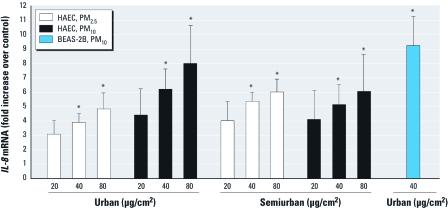
Exposure to PM increases levels of *IL‑8* mRNA. HAECs and BEAS-2B cells were exposed to the indicated size fractions and concentrations of freshly suspended Mexicali PM or to media alone for 4 hr. Levels of *IL‑8* mRNA in cell lysates were measured using RT-PCR (TaqMan; Applied Biosystems, Foster City, CA, USA), normalized to levels of *GAPDH* mRNA, and are expressed as fold increases over media control. Data are mean ± SE (*n* = > 3). *p < 0.05.

*Exposure to PM induces* IL-8 *gene expression in HAECs.* To determine the relative inflammatory potency of PM, we measured the expression of the proinflammatory gene *IL-8* in HAECs exposed to a range of concentrations of PM_10_ and PM_2.5_ collected from urban and semiurban locations in Mexicali, Mexico (Osornio-Vargas et al. 2010). Levels of *IL-8* mRNA increased in a dose-dependent manner after exposure to each particle type (Figure 1). Exposure to 40 or 80 μg/cm^2^ of all PM types for 4 hr resulted in 3.9- to 8-fold increases in *IL-8* mRNA that were statistically significantly different from untreated controls. Incubation of PM_10_ with the lipopolysaccharide (LPS)-binding polypeptide polymyxin B did not change its potency in inducing *IL-8* expression. Moreover, addition of LPS up to 1 μg/mL had no effect on *IL-8* mRNA levels in HAECs (data not shown). We observed no statistically significant differences in potency between the various types/sources of PM; thus, we selected PM_10_ from an urban location for further study. A comparison of HAECs and the BEAS-2B human bronchial epithelial cell line showed similar responses to PM_10_ exposure and established the suitability of BEAS-2B cells as a surrogate for HAECs in this study (Figure 1).

**Figure 2:**
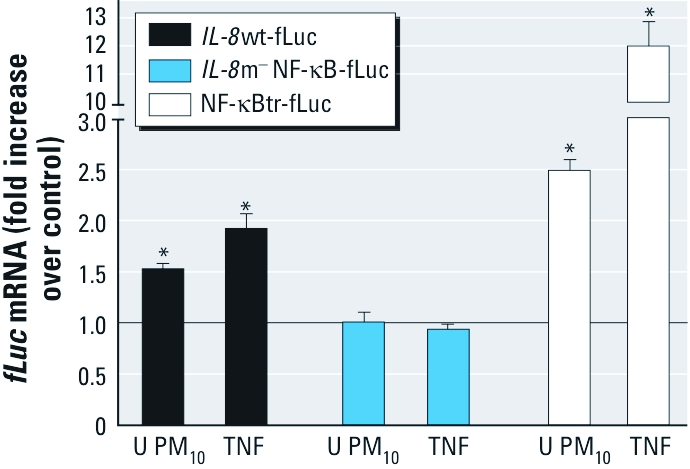
PM-induced *IL‑8* transcription is NF-κB dependent. BEAS-2B cells were transduced with lentiviral vectors encoding *IL‑8*wt-fLuc, *IL‑8*m^–^NF-κB-fLuc, or NF-κBtr-fLuc before exposure to urban (U) PM_10_ at 40 μg/cm^2^ for 4 hr, TNFα (20 ng/mL) for 15 min, or media alone. Promoter activity was assessed by measuring levels of *fLuc* mRNA by RT-PCR. *fLuc* mRNA levels were normalized to *EGFP* mRNA levels, and results are expressed as fold change over untreated controls. Data are mean ± SE (*n* = 3). *p < 0.05.

*PM_10_-induced* IL-8 *expression involves NF-*κ*B–dependent transcriptional activity.* To identify the molecular mechanism responsible for PM-induced *IL-8* expression, we measured the transcriptional activity of *IL-8* wild-type (*IL-8*wt-fLuc) and *NF-*κ*B* mutant (*IL-8*m^–^NF-κB–fLuc) reporter constructs expressed in BEAS-2B cells exposed to PM_10_ using TNFα as a positive control for comparison. Preliminary experiments showed that the presence of particles interfered with optical measurements of luminescence and fluorescence. Therefore, we used *fLuc* and *EGFP* mRNA levels measured by RT-PCR as readouts of the promoter activity. Exposures to 40 μg/cm^2^ PM_10_ for 4 hr resulted in statistically significant increases in promoter reporter activity in BEAS-2B cells expressing the human wild-type *IL-8* promoter. In contrast, BEAS-2B cells expressing an *IL-8* promoter with a mutated NF-κB response element showed no response to the same exposures (Figure 2).

As an independent measurement of NF-κB–dependent transcriptional activity, we next used PM_10_ to stimulate BEAS-2B cells transduced with a lentiviral vector encoding a luciferase reporter gene driven by a concatenated NF-κB consensus sequence (NF-κBtr-fLuc). Exposure to PM_10_ stimulated a statistically significant increase in NF-κB–dependent transcriptional activity in BEAS-2B cells (Figure 2).

**Figure 3:**
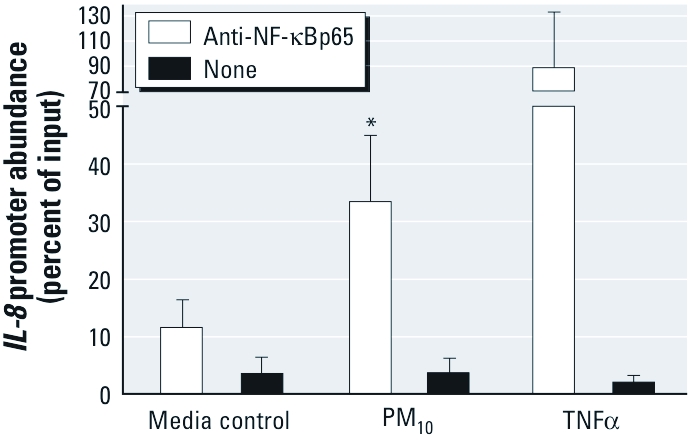
PM_10_ induces NF-κB binding to the *IL‑8* promoter in HAECs. HAECs were exposed to PM_10_ (40 μg/cm^2^) for 4 hr, TNFα (20 ng/mL) for 15 min, or media alone. ChIP followed by RT-PCR was used to measure p65 binding to genomic *IL‑8* promoter sequences. Data are mean ± SE (*n* = 3). *p < 0.05.

*Exposure to PM_10_ induces p65 binding to the NF-*κ*B response element in the* IL-8 *promoter.* Next we investigated whether PM_10_-induced *IL-8* transcriptional activity is accompanied by a corresponding increase in transcription factor binding to NF-κB response elements in the native *IL-8* promoter in HAECs exposed to PM_10._ ChIP analysis using an antibody directed to p65 followed by RT-PCR amplification of immunoprecipitated *IL-8* promoter DNA sequences containing the NF-κB response elements showed that PM_10_ exposure resulted in a marked increase in p65 binding to the genomic *IL-8* promoter in HAEC relative to media-exposed controls (Figure 3). As expected, treatment with TNFα induced a comparatively large increase in immunoprecipitated *IL-8* promoter DNA in HAECs.

**Figure 4:**
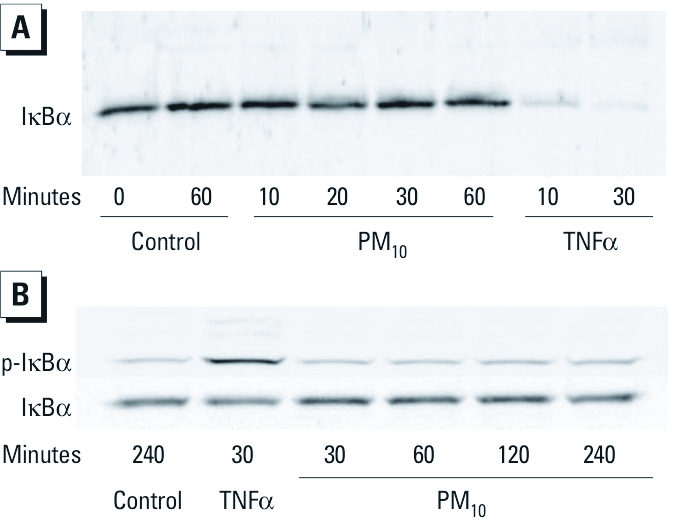
PM-induced NF-κB activation does not involve the canonical pathway in HAECs. (*A*) HAECs were exposed to PM_10_ (40 μg/cm^2^), TNFα (20 ng/mL), or media alone for 10–60 min, and lysates were subjected to Western blotting using anti-IκBα antibody. (*B*) HAECs were pretreated for 30 min with 20 μM of proteosomal inhibitor MG-132 and exposed to PM_10_ (40 μg/cm^2^) or media alone for 30–240 min or TNFα (20 ng/mL) for 30 min; cell lysates were immunoblotted using anti-p‑IκBα and anti-IκBα antibodies. Data are representative of three or more experiments.

*Exposure to PM_10_ induces NF-*κ*B–dependent transcriptional activity that does not involve I*κ*B*α *phosphorylation and degradation.* Phosphorylation and degradation of IκBα are hallmark events in the activation of the canonical NF-κB pathway. Therefore, we examined the effect of PM_10_ exposure on the phosphorylation and abundance of IκBα in HAECs. Western blotting analysis showed that acute challenge with TNFα induced a significant and rapid decrease in the intracellular concentration of IκBα in HAECs (Figure 4A). In contrast, we observed no changes in levels of IκBα in HAECs exposed to 40 μg/cm^2^ PM_10_ for up to 1 hr. Similarly, we detected an increase in the level of p-IκBα in response to TNFα treatment, yet exposure to PM_10_ did not induce a change in p-IκBα levels in HAECs at any of the exposure time points examined (Figure 4B). These results indicated that PM_10_-induced NF-κB transcriptional activity does not involve the canonical activation pathway.

**Figure 5:**
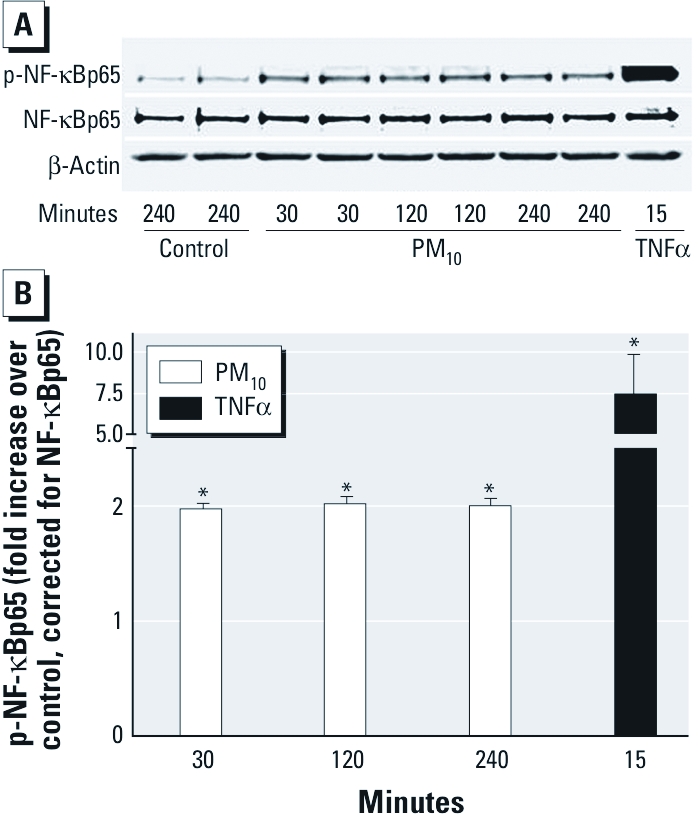
Exposure to PM_10_ induces NF-κB activation through an alternate pathway in HAECs. (*A*) Cell lysates from HAECs exposed to PM_10_ (40 μg/cm^2^) for 30 min to 4 hr or TNFα (20 ng/mL) for 15 min were immunoblotted using antibodies to p-NF-κBp65 (Ser 536), NF-κBp65 (Ser 536), and β-actin. Results are representative of three separate experiments. (*B*) Densitometric quantitation of p-p65 band optical density. Data are mean ± SE (*n* = 3). *p < 0.05.

*PM_10_-induced transcriptional expression of* IL-8 *is dependent on phosphorylation of p65.* Phosphorylation of the NF-κB subunit p65 at multiple serine sites has been reported to increase NF-κB–dependent transcriptional activity in an IκBα-independent manner in a number of cell types (Neumann and Naumann 2007). Therefore, we investigated whether NF-κB activation in HAECs exposed to PM_10_ occurs through phosphorylation of p65. Densitometry of Western blots showed a statistically significant 2-fold increase in levels of phosphorylated p65 (serine 536) after 30 min of exposure to 40 μg/mL PM_10_ that remained elevated for up to 4 hr of exposure (Figure 5).

**Figure 6:**
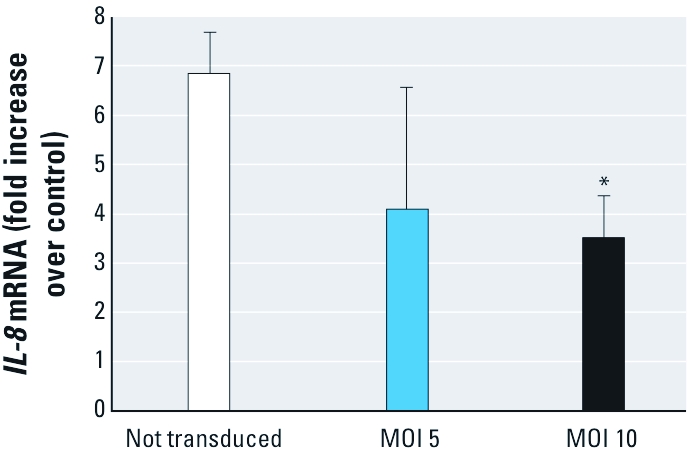
*IL‑8* transcription induced by PM_10_ requires p65 phosphorylation. BEAS-2B cells transduced with a lentiviral vector encoding pTRED-CMV_p65 at an MOI of 5 or 10, in which serines 276, 529, and 536 were mutated to alanines, were exposed to PM_10_ (40 μg/cm^2^) or media alone for 4 hr. Levels of *IL‑8* mRNA in lysates were measured using RT-PCR, normalized to levels of *GAPDH* mRNA, and expressed as fold increases over media control. Data are mean ± SE (*n* = 3). *p < 0.05, compared with the untransduced control.

We next investigated the functional link between p65 phosphorylation and the induction of *IL-8* transcription in HAECs exposed to PM_10_. BEAS-2B cells were transduced with a retrovirus expressing a dominant negative p65 in which three major phosphorylation sites were eliminated by substituting alanines for serines 276, 529, and 536. RT-PCR analyses showed that, relative to control cells, PM_10_-induced *IL-8* expression was blunted in BEAS-2B cells in which p65 serine phosphorylation was prevented (Figure 6).

## Discussion

Although the association of ambient air PM exposure with cardiopulmonary health effects is well established (Peters et al. 2001; Wellenius et al. 2005), the mechanisms by which inhaled PM exerts such deleterious effects remain unclear. As a primary target of inhaled pollutants, the airway epithelium is capable of initiating or supplementing pulmonary inflammatory defenses by synthesizing a number of mediators that can recruit and activate inflammatory cells, thereby promoting inflammation that is thought to culminate in cardiovascular dysfunction (Bai et al. 2007). Our group has reported inflammatory mediator expression by HAECs exposed to a variety of PM components, including diesel exhaust (Tal et al. 2010), zinc ions (Kim et al. 2006; Wu et al. 2010), and ultrafine elemental carbon (Kim et al. 2005). In the present study, we enhance the environmental relevance of these observations by showing that a “real-world” PM sample induces *IL-8* expression through activation of a specific physiological pathway involving an alternate NF-κB activation mechanism.

Several studies have established phosphorylation of p65 as an alternate mechanism leading to the activation of NF-κB–dependent transcriptional activity in response to physiological stimuli such as growth factors, gasotransmitters, and cytokines (Bijli et al. 2008; Chantôme et al. 2004; Kai et al. 2009; Reber et al. 2009; Zhang et al. 2005). p65 phosphorylation is also involved in NF-κB activation induced by LPS, oncogenic viral infection, and cellular transformation (Chen and Harrison 2005; Doyle et al. 2005; Hu et al. 2004). The mechanisms of action of silica (Kang et al. 2006) and the peroxisome proliferator-activated receptor-γ ligand ciglitazone (Chen and Harrison 2005), both xenobiotics that induce NF-κB activation, have been shown to include p65 phosphorylation. In addition, we previously reported that divalent zinc induces *IL-8* expression through an NF-κB–dependent mechanism that involves serine-specific phosphorylation of p65 in BEAS-2B cells (Kim et al. 2007). However, in contrast to the data we show in the present study, zinc also induced IκBα phosphorylation, albeit without inducing its degradation (Kim et al. 2007). We have also reported that ultrafine carbon particles induce *IL-8* expression in HAECs through a mechanism that involves p38 activation, without involvement of NF-κB (Kim et al. 2005). It is possible that the signaling difference seen between ultrafine carbon particles and Mexicali PM is evidence that multiple mechanisms exist in HAECs for the induction of *IL-8* expression in response to a broad range of inhaled xenobiotic materials. It is noteworthy that oxidant stress is a critical event in the toxicity of each of these agents (Atarod and Kehrer 2004; Beck-Speier et al. 2005; Ghio et al. 1992).

Oxidant-induced loss of signaling quiescence, possibly involving inhibition of phosphatases that oppose the activating effect of kinases that phosphorylate p65, is a common feature in the toxicity of diverse groups of chemical agents (Samet and Tal 2010). Therefore, although the upstream mechanisms that initiate p65 phosphorylation induced by PM_10_ have not been identified, it is possible to hypothesize that an oxidant-dependent event is involved. Moreover, although a ligand-mediated activation of a receptor-initiated signaling event cannot be ruled out as a contributing mechanism, the prolonged time course of Mexicali-induced NF-κB activation and *IL-8* expression is consistent with a permissive process such as phosphatase inhibition as an initiating signaling event.

A diverse array of kinases has been implicated in the phosphorylation of p65, including IKKβ (IκB kinase β) (Kang et al. 2006), PKCζ (protein kinase Cζ) (Kai et al. 2009), Btk (Bruton’s tyrosine kinase) (Doyle et al. 2005), CKII (casein kinase II) (Chantôme et al. 2004), Rsk1 (ribosomal S6 kinase 1) (Bohuslav et al. 2004), Syk (spleen tyrosine kinase) (Bijli et al. 2008), MSK1 (mitogen- and stress-activated protein kinase 1) (Reber et al. 2009), and PI3K (phosphoinositide 3-kinase) (Liu et al. 2008). We have observed that pharmacological inhibition of PI3K failed to block PM_10_-induced *IL-8* expression, implying that the PI3K-phosphatase and tensin homolog (PTEN) pathway is not involved. Separately, we also determined that inhibition of MEK (MAP kinase-ERK kinase), JNK (Jun-N-terminal kinase), p38 kinase, and the src tyrosine kinases p56^lck^, p59^fynT^, and Hck failed to block PM_10_-induced *IL-8* expression (Silbajoris R, unpublished data). Given the number of pathways that are known to lead to p65 phosphorylation, our finding that PM_10_-induced gene expression does not involve canonical activation of NF-κB will require detailed follow-up study in order to identify the upstream kinase(s) involved, as well as the proximal event that leads to p65 phosphorylation in response to PM_10_. Such studies will expand the results presented here and further elucidate the mechanisms through which inflammatory responses are regulated in the airway epithelium after inhalation of PM_10_.

The PM_10_ sample we used in this study was obtained from Mexicali, Mexico, a semirural border city afflicted with high levels of inhalable PM derived from both combustion and geological sources. Previous studies on Mexicali PM have associated particle size with specific chemical composition, source, and biological effects (Mendoza et al. 2010; Osornio-Vargas et al. 2010). However, in the present study, PM_2.5_ and PM_10_ collected in urban and semiurban locations in Mexicali were of comparable potency in inducing *IL-8* expression in HAECs. Further, pharmacological inhibition of NF-κB activation abrogated *IL-8* expression induced by each of the Mexicali particles used in this study (Silbajoris R, unpublished data). These results suggest that a property these particles have in common is responsible for their inflammogenicity.

Considering that these materials were collected from ambient air, it is not surprising that analyses showed significant endotoxin levels associated with each of the Mexicali particles, raising the possibility that LPS contributes to their bioactivity. However, endotoxin activates the canonical NF-κB pathway, and we did not observe such activation with the Mexicali particles. Moreover, HAECs are known to express very low levels of endotoxin receptors (Becker et al. 2000), and we have previously reported that endotoxin levels far in excess of those presented to the cells in this study resulted in only minimal induction of *IL-8* expression by HAECs (Silbajoris et al. 2009). In the present study, we confirmed those findings and determined that an endotoxin-binding polypeptide (polymyxin B) does not affect the potency of PM_10_ in inducing HAEC *IL-8* gene expression. Thus, the toxicity of Mexicali PM is attributable to other chemical components associated with these particles. Of the metals that are present in Mexicali PM_10_ [see Supplemental Material, Table 1 (http://dx.doi.org/10.1289/ehp.1103594)], only zinc has been reported to induce NF-κB activation that involves p65 phosphorylation (Kim et al. 2007), albeit at concentrations considerably higher than the submicromolar concentrations used in the present study.

Although the considerable chemical and physical complexity that characterizes ambient PM prevents a ready identification of bioactive components and sources in this mechanistic study, the results presented here nonetheless provide insight into the contribution of proinflammatory processes in the lung to the toxicity of PM inhalation. Further, these findings lend biological plausibility in support of epidemiological reports of the adverse public health impact of PM exposure.

## Supplemental Material

(96 KB) PDF
