# Supplementary material for: Ambient Particulate Matter Induces Interleukin-8 Expression through an Alternative NF-κB (Nuclear Factor-Kappa B) Mechanism in Human Airway Epithelial Cells
Source: Environ Health Perspect. 2011 Jun 10;119(10):1379–83. doi: 10.1289/ehp.1103594 (PMC3230452; doi:10.1289/ehp.1103594)
Supplement: (96 KB) PDF [file ehp.1103594.s001.pdf]

SUPPLEMENTAL MATERIAL

Ambient Particulate Matter Induces IL-8 Expression Through an alternative NF-κB Mechanism in Human Airway Epithelial Cells

Robert Silbajoris[1], Alvaro R. Osornio-Vargas[2*], Steven O. Simmons[3], William Reed[4], Philip A. Bromberg[4], Lisa A. Dailey[1] and James M. Samet[1]

[1]Environmental Public Health Division and [3]Integrated Systems Toxicology Divisions, National Health and Environmental Effects Research Laboratory, U.S. Environmental Protection Agency, Research Triangle Park, NC, USA, [2]Instituto Nacional de Cancerologia, Mexico City, Mexico, [4]Center for Environmental Medicine, Asthma and Lung Biology, University of North Carolina at Chapel Hill, Chapel Hill, NC, USA

METHODS

PM collection and composition. PM were sampled at two monitoring stations in Mexicali, Mexico using High Volume samplers (Tisch TE607OV, Roswell, GA) and modified nitrocellulose membranes (Sartorius 11302-131, Satelite, EdeM, Mexico) during 24 h periods on alternate days at least twice a week from October 2005 to March 2006. PM samples were mechanically recovered from the membranes and pooled by month, site and particle size and analyzed for elemental composition by particle induced X-ray emission (Table 1). Using a Malvern Zetasizer (Malvern, UK), the mean diameter of the UPM_{10} particles was determined to be 1.6 microns.

Plasmid construction. Lentiviral transfer plasmids for human IL-8 wild-type (IL-8wt) and NF-κB-site mutant (IL-8m^{-} NF-κB) luciferase reporters, as well as the NF-κB tandem repeat (NF-κBtr) luciferase reporter have been previously described (Tal et al. 2010). A lentiviral transfer plasmid encoding an enhanced green fluorescent protein (EGFP) reporter under control of the human EF1α promoter (pTZV-EF1a_EGFP) was obtained from Open Biosystems (Huntsville, AL). A cDNA encoding a mutated human RELA (p65) protein (S276A/S529A/S536A triple mutant) under the control of the cytomegalovirus promoter (CMV) was isolated from pFLAG-p65 triple mutant (Kim et al. 2007) by restriction digest with SpeI and BamHI. The resulting fragment was subcloned into a modified pTZV lentiviral transfer plasmid (Open Biosystems, Huntsville, AL) between the NheI and BamHI restriction sites. The recombinant transfer vector pTRED-CMV_p65 triple mutant was verified by fluorescent DNA capillary sequencing.

Lentiviral vector production and titering. HEK293T cells were co-transfected in 10 cm dishes with purified IL-8wt-luciferase, IL-8m$^-$ NF-κB-luciferase, NF-κBtr-luciferase, pTZV-F1α_EGFP or pTRED-CMV_p65 triple mutant transfer vector plasmids and lentiviral packing mix (Open Biosystems; Huntsville, AL) according to manufacturer's instructions. Sixteen hours post-transfection, cell culture medium was replaced with 12 ml fresh DMEM and cells were incubated for an additional 48 h at 37°C. Medium was then harvested and detached cells were pelleted by centrifugation for 10 minutes at 5,000*g*. The resulting supernatants from the individual transfections were concentrated once by low-speed centrifugation through an Amicon Ultra 100kD centrifuge filter unit (Millipore; Billerica, MA), and the retentates were aliquoted and stored at -80°C. To determine viral titers, 50,000 HEK293T cells stably expressing the TetOff (rtTA3; Clontech, Mountain View, CA) transactivator were transduced with 50 µl of lentiviral stock dilutions ranging from 1:10 to 1:781,250. Viral titers for IL-8wt-luciferase, IL-8m$^-$ NF-κB-luciferase, NF-κBtr-luciferase, or pTRED-CMV_p65 triple mutant (expressed as transducing units per ml viral stock) were determined 96 hours post-transduction by counting red fluorescent colonies by fluorescent microscopy (red colonies form due to rTTA3-mediated activation of the secondary tetracycline-inducible turboRED fluorescent reporter) and multiplying the colony count by the dilution and volume factors. EF1α_EGFP vector was titered in the same manner using green fluorescent protein (encoded by the vector) to quantify colonies.

RT-PCR. Relative gene expression in HAEC and BEAS-2B cells was quantified using RT-PCR. Total RNA was isolated using a kit (Qiagen, Valencia, CA) and cDNA was generated using a reverse transcription kit (Applied Biosystems, Foster City, CA). Oligonucleotide primer pairs and dual-labeled fluorescent probes (Table 1) for IL-8, GAPDH, firefly luciferase/pGL3 (fLuc)

and enhanced green fluorescent protein (EGFP) were obtained from IDT (Coralville, IA). Quantitative fluorogenic amplification of cDNA was performed using the ABI Prism 7500 Sequence Detection System, primer/probe sets of interest and TaqMan Universal PCR Master Mix (Applied Biosystems). The relative abundance of mRNA levels of IL-8 and GAPDH, fLuc, and EGFP was determined from standard curves generated from serially diluted pools of cDNA prepared from HAEC, pGL3-basic plasmid (Promega, Madison, WI) or pHygroEGFP plasmid (Clonetech, Mountain View, CA), respectively. Levels of GAPDH mRNA were used to normalize levels of IL-8 mRNA. To control for transduction efficiency, the relative abundance of EGFP mRNA was used to normalize levels of fLuc mRNA. Results are expressed as fold change over media controls.

Supplemental Material, Table 1. Elemental concentrations found in Mexicali urban PM_{10} samples, expressed as µg per mg of particle mass and as a percent of the analytes shown.

	**µg/mg**	**%**
**Si**	5.55	32.3
**Ca**	3.91	22.7
**Al**	2.04	11.9
**Fe**	1.60	9.33
**K**	1.33	7.71
**S**	0.72	4.20
**Mg**	0.57	3.32
**Cl**	0.40	2.32
**Ti**	0.24	1.41
**P**	0.21	1.19
**Zn**	0.18	1.04
**V**	0.13	0.76
**Cr**	0.12	0.68
**Cu**	0.09	0.51
**Mn**	0.07	0.39
**Ni**	0.03	0.18
**Total**	17.20	100

Supplemental Material, Table 2. Sequences of oligonucleotide primers and dual labeled probes (5' FAM/3' TAMRA) used in RT-PCR.

**Gene**		**Sequence (5'/3')**
EGFP	forward primer	CTGCTGCCCGACAACCA
	reverse primer	TGTGATCGCGCTTCTCGTT
	probe	TACCTGAGCACCCAGTCCGCCCT
fLuc/pGL3	forward primer	CCGCCTGAAGTCTCTGATTAAGTAC
	reverse primer	TGGAGCAAGATGGATTCCAAT
	probe	CAGCGGGAGCCACCTGATAGCCTT
GAPDH	forward primer	GAAGGTGAAGGTCGGAGTC
	reverse primer	GAAGATGGTGATGGGATTTC
	probe	CAAGCTTCCCGTTCTCAGCC
IL-8	forward primer	TTGGCAGCCTTCCTGATTTC
	reverse primer	TATGCACTGACATCTAAGTTCTTTAGCA
	probe	CCTTGGCAAAACTGCACCTTCACACA

REFERENCES

Kim YM, Cao D, Reed W, Wu W, Jaspers I, Tal T, et al. 2007. Zn2+-induced NF-kappaB-dependent transcriptional activity involves site-specific p65/RelA phosphorylation. Cell Signal 19(3): 538-546.

Tal TL, Simmons SO, Silbajoris R, Dailey L, Cho SH, Ramabhadran R, et al. 2010. Differential transcriptional regulation of IL-8 expression by human airway epithelial cells exposed to diesel exhaust particles. Toxicol Appl Pharmacol 243(1): 46-54.
